# Transcription Factor Nrf2 Modulates Lipopolysaccharide-Induced Injury in Bovine Endometrial Epithelial Cells

**DOI:** 10.3390/ijms241311221

**Published:** 2023-07-07

**Authors:** Pengjie Song, Chen Liu, Mingkun Sun, Jianguo Liu, Pengfei Lin, Huatao Chen, Dong Zhou, Keqiong Tang, Aihua Wang, Yaping Jin

**Affiliations:** Key Laboratory of Animal Biotechnology of the Ministry of Agriculture, College of Veterinary Medicine, Northwest A&F University, Xianyang 712100, China; songpengjie@nwafu.edu.cn (P.S.);

**Keywords:** bovine endometritis, endoplasmic reticulum stress, inflammation, Nrf2

## Abstract

Endometritis in high-yield dairy cows adversely affects lactation length, milk quality, and the economics of dairy products. Endoplasmic reticulum stress (ERS) in bovine endometrial epithelial cells (BEECs) occurs as a consequence of diverse post-natal stressors, and plays a key role in a variety of inflammatory diseases. Nuclear-factor-erythroid-2-related factor 2 (Nrf2) is an important protective regulatory factor in numerous inflammatory responses. However, the mechanism by which Nrf2 modulates inflammation by participating in ERS remains unclear. The objective of the present study was to explore the role of Nrf2 in lipopolysaccharide (LPS)-induced injury to BEECs and to decipher the underlying molecular mechanisms of this injury. The expression of Nrf2- and ERS-related genes increased significantly in bovine uteri with endometritis. Isolated BEECs were treated with LPS to stimulate the inflammatory response. The expression of Nrf2 was significantly higher in cells exposed to LPS, which also induced ERS in BEECs. Activation of Nrf2 led to enhanced expression of the genes for the inflammation markers *TNF-α*, *p65*, *IL-6*, and *IL-8* in BEECs. Moreover, stimulation of Nrf2 was accompanied by activation of ERS. In contrast, Nrf2 knockdown reduced the expression of *TNF-α*, *p65*, *IL-6*, and *IL-8*. Additionally, Nrf2 knockdown decreased expression of ERS-related genes for the GRP78, PERK, eIF2α, ATF4, and CHOP proteins. Collectively, our findings demonstrate that Nrf2 and ERS are activated during inflammation in BEECs. Furthermore, Nrf2 promotes the inflammatory response by activating the PERK pathway in ERS and inducing apoptosis in BEECs.

## 1. Introduction

Bovine endometritis involves inflammation of the endometrium, which decreases fertility and reproduction in dairy cows, thereby causing significant economic losses in agriculture [1]. Endometritis, a major cause of mare infertility arising from failure to remove bacteria, spermatozoa, and inflammatory exudate post breeding, is often undiagnosed in advance [2]. Cows affected by endometritis showed significantly decreased conception rates, prolonged days to first service and days open, as well as a reduced number of cows pregnant [3]. Bacterial infection, which often occurs after calving, is the most common cause of bovine endometritis, although inadequate hygiene during insemination, poor nutrition, and genetic factors also may contribute to development of the condition [4]. The molecular mechanisms that underpin the response to bovine endometritis are complex and involve numerous regulatory factors, but the precise pathways that are involved remain unclear.

The endoplasmic reticulum (ER) is a key organelle that is implicated in diverse cellular processes, including protein synthesis, modification, integration, folding, and quality control, calcium storage and homeostasis, and lipid biosynthesis [5]. When external stimuli perturb the equilibria of these processes in the ER, protein synthesis signals are disrupted, which induces the aggregation of unfolded or misfolded proteins. ER homeostasis is unbalanced as a consequence, which ultimately leads to ER stress (ERS) [6]. ERS and oxidative stress are activated in response to numerous stressors [7]. ERS plays a key role in the pathophysiology of cerebral ischemia as excessive or long-term ERS causes activation of apoptotic pathways [8]. Moreover, ERS is implicated in numerous inflammatory diseases [9]. Furthermore, a prolonged state of ERS not only affects uterine involution but also supports the appearance of endometritis in bovine endometrial epithelial cells (BEECs) during the postpartum period of dairy cows [10].

The unfolded protein response (UPR) is triggered in response to damage caused by ERS and clears misfolded proteins from the ER, thereby delaying the progression of ERS [11]. UPR principally involves the PERK-eIF2α, IRE1-XBP1, and ATF6 signal transduction pathways [12]. Protein kinase R-like ER kinase (PERK), activating transcription factor 6 (ATF6), and inositol-requiring enzyme 1 (IRE1) are maintained in inactive states when bound under normal physiological conditions to the ER molecular chaperone, glucose-regulated protein 78 (GRP78). However, these proteins are released from GRP78 and activated during stress [13]. Activated PERK is phosphorylated and blocks eukaryotic translation initiation factor-2α (eIF2α), which inhibits protein synthesis [14]. In contrast, activated IRE-1 modifies the mRNA for X-box binding protein-1 (XBP-1) to produce an isoform of XBP1 that drives the UPR gene expression program. After disassociation from GRP78, ATF6 is cleaved by proteases S1P and S2P in the Golgi apparatus [14]. The truncated version of ATF6 regulates the expression of ERS-specific genes [15]. UPR reduces the aggregation of unfolded or misfolded proteins during ER stress by the preceding signaling pathways, alleviates the cellular damage caused by accumulated proteins, restores ER homeostasis, and enables stressed cells to survive [6]. In addition, the PERK protein activates the ATF4 and IRE1 pathways during ERS-induced apoptosis and following initiation of the UPR [8]. The activation of IRE1α-dependent apoptosis promotes formation of the spliced form of XBP that mediates proapoptotic signaling, which in turn enhances cell apoptosis and inhibits cell survival factors.

Nuclear-factor-erythroid-2-related factor 2 (Nrf2) is a transcription factor of the Cap’n’Collar basic leucine zip family. The protein comprises 605 amino acids and maintains cell redox homeostasis and exerts anti-inflammatory functions [16]. The activity of Nrf2 is regulated by multiple mechanisms, including the ubiquitin-proteasome degradation system, post-translational modifications, epigenetic regulation, and autoregulation. Nrf2 alleviates inflammation through different mechanisms [17]. The first mechanism depends on reactive oxygen species (ROS), which are important mediators of tissue damage and which stimulate the production of inflammatory cytokines [18]. Research demonstrated that activation of the Nrf2/Keap1 pathway by lead impaired oocyte maturation and fertilization by inducing oxidative stress, leading to a decrease in the fertility of female mice [19]. Activation of the Nrf2/HO-1 signaling pathway by melatonin could protect follicular integrity [20]. However, activation of Nrf2 also induces the expression of antioxidant genes and reduces intracellular ROS levels, thereby providing protective effects against inflammation [21]. A previous study has shown that activating the Nrf2/HO-1 pathway by platelet-rich plasma significantly inhibited endometrial cell injury and alleviated the inflammatory response [22]. Nrf2 activation by andrographolide was able to protect against the LPS-induced inflammatory response in bovine endometrial epithelial cells [23]. However, little is known about the mechanism of action of Nrf2 in ERS-mediated bovine endometritis. Therefore, the present study aimed to investigate the role and molecular mechanisms of Nrf2 in this disease.

## 2. Results

### 2.1. Nrf2 Expression Is Increased Significantly in Endometriotic Uteri and Is Associated with ERS and UPR

**Figure 1:**
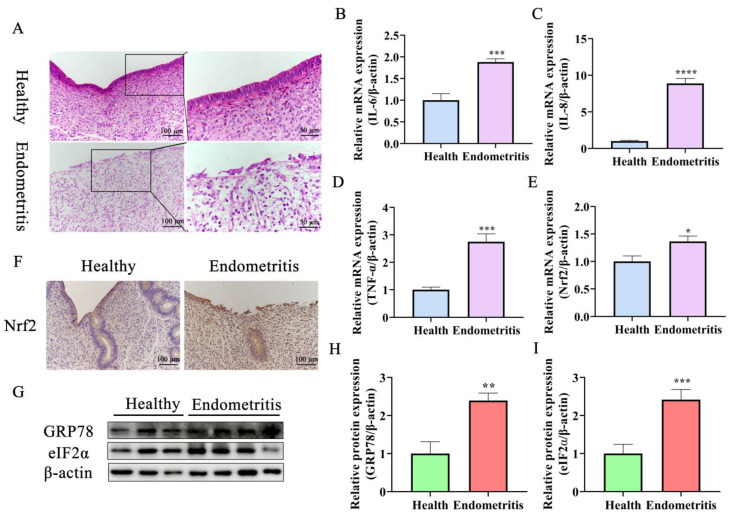
ERS and increased Nrf2 expression occurred in bovine uterine tissues with endometritis. (**A**) Histopathological characterization of samples from the uteri of healthy (Health) and endometriotic (Endom) dairy cows (Bar = 50 μm). (**B**–**D**) The expression of inflammation-related factors *IL-6*, *IL-8*, and *TNF-α* in uterine tissue was assessed by RT-qPCR. (**E**) The mRNA expression of *Nrf2* was detected in uteri with and without endometritis by RT-qPCR. (**F**) Immunohistochemical analysis was used to visualize Nrf2 expression in dairy cow uteri (Bar = 100 μm). (**G**–**I**) Western blotting was used to analyze GRP78 and eIF2α levels in dairy cow uteri. The experiments were repeated in triplicate and expression data were normalized to those of *β-actin*. The unpaired Student’s *t*-test was used to compare the results between the healthy and endometritis groups. Statistical significance was set at *p* < 0.05 (* *p* < 0.05, ** *p* < 0.01, *** *p* < 0.001, **** *p* < 0.0001).

Uteri of dairy cows were divided into healthy and endometritis groups based on hematoxylin–eosin (HE) staining and the expression of inflammatory factors (Figure 1). Epithelial cells in the endometriotic tissues were exfoliated with a large number of inflammatory cells infiltrating the lamina propria of the uterus compared with the healthy group (Figure 1A). Moreover, the expression of inflammatory cytokines *IL-6*, *IL-8*, and *TNF-α* was increased significantly compared with the unaffected tissue (Figure 1B–D). The expression of Nrf2 was also greater in the endometriotic samples compared with the healthy samples (Figure 1E). Additionally, the expression of ERS marker protein GRP78 was higher in tissues with endometritis compared with healthy tissues (Figure 1G,H). Finally, the expression of UPR-signaling-pathway-related protein eIF2α was increased significantly in uteri with endometritis compared to healthy uteri (Figure 1G,I). Overall, these results suggest that ERS is implicated in dairy cow endometritis and that the Nrf2 transcription factor is involved in regulation of the disease.

### 2.2. Lipopolysaccharide Activates the Inflammatory Response and ERS in Bovine Endometrial Epithelial Cells

**Figure 2:**
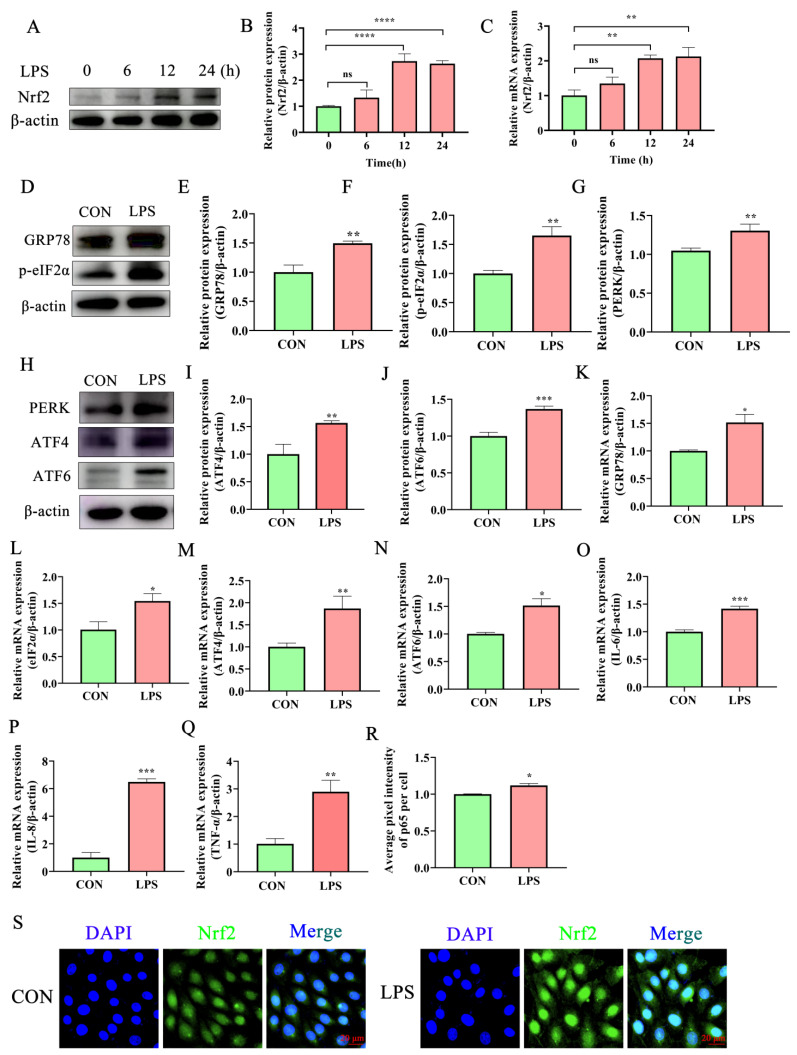
LPS-induced inflammatory response and ERS response in BEECs. (**A**–**C**) Western blot and RT-qPCR analysis of Nrf2 expression in BEECs treated with LPS. (**D**–**J**) Western blot analysis of the expression of ERS-related proteins (GRP78, eIF2α, PERK, ATF4, and ATF6) in BEECs with and without LPS treatment. (**K**–**N**) The mRNA levels of *GRP78*, *eIF2α*, *ATF4*, and *ATF6* in BEECs with and without LPS treatment were assessed by RT-qPCR. (**O**–**Q**) The mRNA levels of inflammatory cytokines *IL-8* and *IL-10* in BEECs with and without LPS treatment were assessed by RT-qPCR. (**R**,**S**) Immunofluorescence of Nrf2 and DAPI-staining of BEECs with and without LPS treatment (Bar = 20 μm). LPS was used at 10 μg/mL in all cases. One-way ANOVA and Tukey’s post hoc test were used for statistical analysis. Data are the mean ± SEM (*n* = 3). ns no significance, * *p* < 0.05, ** *p* < 0.01, *** *p* < 0.001, **** *p* < 0.0001.

Lipopolysaccharide (LPS) is the main pathogenic factor of Gram-negative bacteria and is used widely to stimulate the development of bovine endometritis in vitro [24,25,26]. The expression of *IL-6*, *IL-8*, and *TNF-α* increased significantly in BEECs treated with LPS compared with untreated cells (Figure 2O–Q). Biomarker proteins of ER stress and UPR were analyzed by Western blot to investigate whether these processes mediate protection against the LPS-induced inflammatory response in BEECs. ERS-related proteins (GRP78, eIF2α, PERK, ATF6, and ATF4) were activated significantly at both mRNA and protein levels after LPS treatment for 12 h (Figure 2D–N). A significant increase in the expression of the transcription factor Nrf2 after treatment with LPS for 12 h was also observed (Figure 2A–C,S). In summary, the enhancement of inflammatory cytokines and ERS-related protein activity indicates that exposure to LPS activates the UPR pathway that helps to alleviate ERS. The chronic or excessive activation of the UPR process may lead to prolonged inflammation.

### 2.3. Knockdown of Nrf2 Reduces Both the LPS-Induced Inflammatory Response and ERS in Bovine Endometrial Epithelial Cells

**Figure 3:**
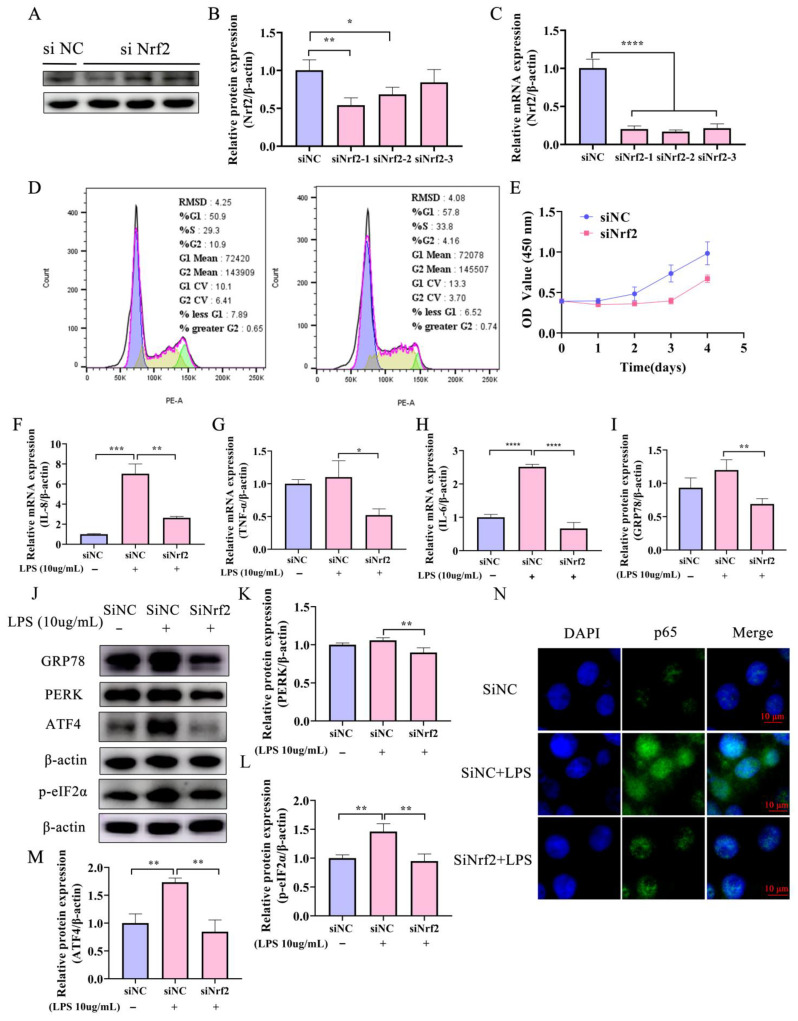
Knockdown of Nrf2 attenuated the LPS-induced inflammatory response and ERS in BEECs. (**A**,**B**) BEECs were transfected with Nrf2 siRNA (siNrf2) or negative control siRNA (siNC) for 24 h and Nrf2 protein expression was detected using Western blot analysis. (**C**) BEECs were transfected with siNrf2 and the mRNA expression of the gene for Nrf2 was assessed by RT−qPCR. (**D**,**E**) Detection of cell cycle using annexin V−FITC and PI staining assay and flow cytometry. (**F**–**H**) BEECs were transfected with siNrf2 and the mRNA expression of inflammatory factors IL−6, IL−8, and TNF−α was assessed by RT−qPCR. (**I**–**M**) BEECs were transfected with siNrf2 or siNC for 24 h and ERS-related protein expression (GRP78, PERK, ATF4, and eIF2α) was detected using Western blot analysis. (**N**) Immunofluorescence analysis of p65 in control and Nrf2 knockdown cells (Bar = 10 μm). Data are presented as mean ± SEM (*n* = 3). One−way ANOVA analysis was used. * *p* < 0.05, ** *p* < 0.01, *** *p* < 0.001, **** *p*< 0.0001.

The role of Nrf2 in the regulation of ERS and inflammation was assessed further using BEECs in which the gene for Nrf2 was knocked down. A small interfering RNA (siRNA) mimic (siNrf2) significantly reduced the expression of Nrf2 at both transcriptional and translational levels (Figure 3A–C). Flow cytometry revealed that cell cycle arrest occurred mainly in G1 and S phases in the knockdown cells compared with the negative control siRNA (siNC) cells (Figure 3D). Moreover, knocking down the expression of Nrf2 inhibited the proliferation of BEECs (Figure 3D,E). RT-qPCR analysis showed that the mRNA levels of inflammatory cytokines *IL-6*, *IL-8*, and *TNF-α* were significantly lower in BEECs transfected with si*Nrf2* and treated with LPS than in cells transfected with siNC and treated with LPS (Figure 3F–H). In addition, immunofluorescence demonstrated that p65 nuclear metastasis was inhibited by interfering with Nrf2 expression during LPS challenge (Figure 3N). These results suggest that Nrf2 interference alleviates the inflammatory response in BEECs induced by LPS.

The siNrf2 sequence reduced production of the GRP78 protein in BEECs treated with LPS (Figure 3H,I) and also inhibited the ERS and apoptosis-related PERK-eIF2α-CHOP pathway indicated by decreased expression of PERK and p-eIF2α proteins (Figure 3I,J,L). Additionally, the level of the ATF4 protein was up-regulated (Figure 3J,M). Collectively, these results suggest that interference with Nrf2 relieves the LPS-induced inflammatory response in BEECs.

### 2.4. Activation of Nrf2 Aggravates the LPS-Induced Inflammatory Response in Bovine Endometrial Epithelial Cells through the ERS PERK Pathway

**Figure 4:**
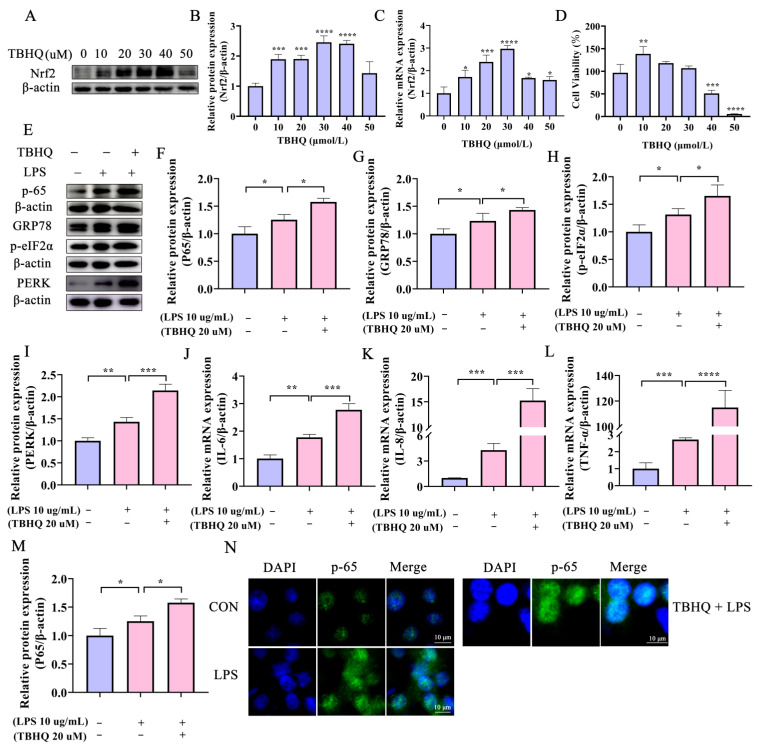
Activation of Nrf2 aggravates the LPS-induced inflammatory response and ERS in BEECs. (**A**–**C**) BEECs were pretreated with the indicated concentrations of TBHQ for 2 h and mRNA and protein levels for Nrf2 were detected by RT−qPCR and Western blotting, respectively. (**D**) Cell viability of BEECs after treatment with different concentrations of TBHQ was assessed by CCK−8 analysis. (**E**–**I**) Western blotting analysis of p65− and ERS−related protein expression in BEECs with or without TBHQ and LPS treatment. (**J**–**L**) RT-qPCR analysis of inflammatory factors in BEECs treated with or without LPS and TBHQ treatment. (**M**,**N**) Immunofluorescence analysis of p65 in BEECs in control and Nrf2−activated cells with or without LPS treatment (Bar = 10 μm). One-way ANOVA analysis was used. Data are the mean ± SEM (*n* = 3). * *p* < 0.05, ** *p* < 0.01, *** *p* < 0.001, **** *p* < 0.0001.

Tert-butylhydroquinone (TBHQ), a metabolite of butylated hydroxyanisole, induces Nrf2 activation [27]. The cytotoxicity of TBHQ in BEECs was investigated by assessing cell viability after treatment with different concentrations of the compound for 2 h. TBHQ at either 20 or 30 μM had no effect on BEEC proliferation (Figure 4D), but significantly improved transcription and translation levels of Nrf2 (Figure 4A–C). Therefore, 20 μM TBHQ was selected for further investigation of the role of Nrf2 in endometritis. Activation of Nrf2 by TBHQ increased the expression of inflammatory cytokines *IL-6*, *IL-8*, and *TNF-α* (Figure 4J–L). In addition, the level of the p65 protein was up-regulated in cells pretreated with TBHQ after LPS challenge (Figure 4E,F). Moreover, TBHQ-mediated activation of Nrf2 intensified the nuclear translocation of p65 (Figure 4M,N) and significantly up-regulated ERS-related proteins after incubation with LPS (Figure 4E,G–I). These combined data indicate that activation of Nrf2 by TBHQ aggravates the LPS-induced inflammatory response in BEECs.

### 2.5. Disorder of Ca^2+^ and CHOP Promote ERS-Mediated Damage in Bovine Endometrial Epithelial Cells

**Figure 5:**
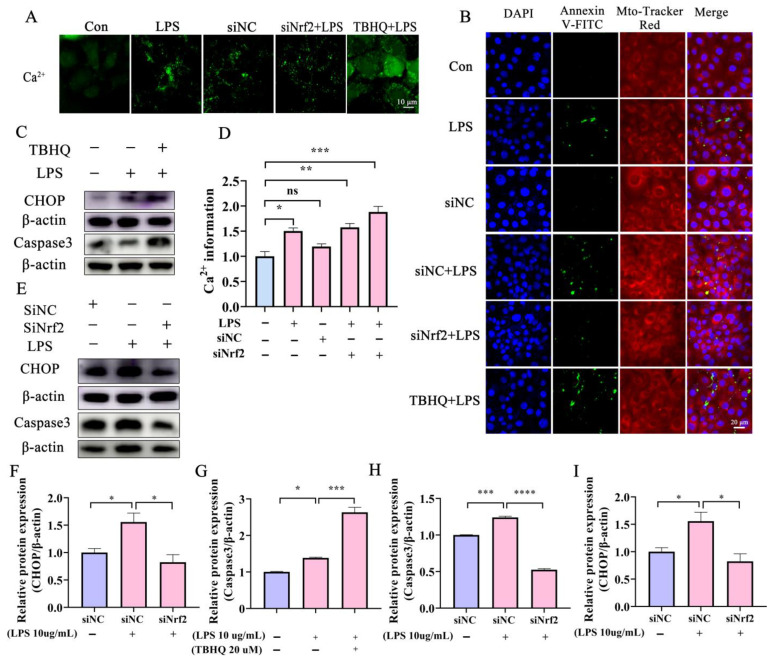
Effects of LPS on Ca^2+^ mobilization and ER function in BEECs. (**A**,**D**) BEECs in control and Nrf2−knockdown cells with or without LPS and TBHQ treatment were assessed for Ca^2+^ efflux from the ER into the cytoplasm using a Ca^2+^ detection kit (Bar = 5 μm). (**B**) Cell apoptosis was analyzed by an apoptosis detection kit with Mito-Tracker Red CMXRos and Annexin V-FITC (Bar = 20 μm). (**C**,**F**,**G**) Apoptosis-related protein expression levels were assessed in Nrf2−activated BEECs treated with or without LPS. (**B**,**E**,**H**,**I**) Western blot was used to analyze apoptosis−related protein expression in Nrf2-knockdown BEECs treated with or without LPS. One−way ANOVA analysis was used. Data are the mean ± SEM (*n* = 3). ns: no significance, * *p* < 0.05, ** *p* < 0.01, *** *p* < 0.001, **** *p* < 0.0001.

The ER is a large, dynamic structure that serves numerous cellular roles, including Ca^2+^ storage, protein synthesis, and lipid metabolism [28]. The impact of different treatments on ER integrity in BEECs was assessed further by detecting cytoplasmic Ca^2+^ levels. LPS administration significantly induced Ca^2+^ efflux from the ER into the cytoplasm (Figure 5A,D). Following Nrf2 knockdown and treatment with LPS, Ca^2+^ efflux into the cytoplasm from the ER decreased significantly compared with cells treated with LPS only (Figure 5A,D). In contrast, Ca^2+^ efflux increased in BEECs that were treated with TBHQ and LPS compared with LPS-only treatment. Ca^2+^ outflow from the ER suggests that the imbalance of Ca^2+^ between the ER and cytoplasm might underpin ERS. The CHOP protein maintains a low level of expression in the cytoplasm under normal conditions, whereas increased CHOP expression indicates ERS. CHOP activates Caspase12, initiates the Caspase cascade, and mediates apoptosis. Both LPS administration and Nrf2 activation with THBQ promoted BEEC apoptosis (Figure 5B). Moreover, activation of Nrf2 increased the expression of LPS-induced apoptosis proteins Caspase3 and CHOP (Figure 5C,F,G). Conversely, knockdown of Nrf2 reduced the expression of these proteins (Figure 5E,H,I). Collectively, these results suggest that activation of Nrf2 promotes the damage of BEECs induced by LPS and induces BEEC apoptosis through the ERS-CHOP pathway.

## 3. Discussion

Endometritis seriously impairs both animal breeding efficiency and the quality of meat and milk in bovine farming. The aim of this study was to probe bovine endometritis by determining the effects of Nrf2 on LPS-induced injury in BEECs. The results demonstrate that Nrf2 exerts a critical role in the pathogenesis of bovine endometritis and is connected with the activation of UPR and ERS in uteri with endometritis. Interestingly, Nrf2 was also activated in LPS-induced mice endometritis [29]. The observations here suggest that Nrf2 may be a potential target for the treatment of dairy cow endometritis.

Nrf2 is a transcription factor that plays a crucial role in regulating cellular antioxidant levels and in detoxification responses [30]. The protein resolves inflammation by modulating the expression of genes that encode antioxidant and anti-inflammatory proteins [31]. Nrf2 activation also reduces the production of ROS and reactive nitrogen species, which are important contributors to the production of inflammatory mediators [32]. Previous studies revealed that inflammatory factors *IL-6* and *TNF-α* were significantly increased in the menstrual blood of women with chronic endometritis [33,34]. Research has shown that activation of Nrf2 and inhibition of NF-κB resulted in a decrease in TLR2/TLR4, which could retard apoptosis and inflammation induced by neisseria gonorrhoeae infection in human endometrial epithelial cells [35]. Similarly, activation of Nrf2 by apigenin could protect against LPS-induced endometritis [36]. Nrf2 activation reduced uterine oxidative stress and inflammation, which are key factors in the development of endometritis [26,37]. A previous study has shown that aucubin activated the Keap1/Nrf2 pathway, promoting the nuclear transfer of Nrf2 and increasing Keap1, Nrf2, HO-1, and NQO1 mRNA and protein levels, and aucubin ameliorates the LPS-induced inflammatory response by inhibiting NF-κB and activating the Keap1/Nrf2 signaling pathway to protect BEECs from LPS induced-damage [38]. Additionally, Nrf2 activation increased the expression of genes involved in the immune response, which helps combat bacterial infections that cause endometritis [39]. Dairy cow primary endometrial epithelial cells were treated with LPS here to stimulate endometritis in vitro. Inflammation elicited by LPS caused an increase in the expression of IL-6, IL-8, and p-65. Concurrently, the expression of Nrf2 was elevated compared with untreated cells. Thus, the inflammatory response and Nrf2 were activated simultaneously in the LPS-induced BEEC inflammatory in vitro model. In addition, the enhanced production of the GRP78, eIF2α, IRE1, and ATF4 proteins following LPS administration demonstrated that UPR and ERS were activated and involved in the inflammatory responses. A recent study showed that meloxicam attenuated the inhibitory effect of the Nrf2 pathway and the phosphorylation levels of p65 and IκBα caused by LPS, and the co-treatment of meloxicam and LPS reduced the content of oxidative stress markers and the mRNA levels of the pro-inflammatory genes, and improved antioxidant enzyme activities and the corresponding gene expression as compared with the cells treated with LPS alone [40]. In summary, the results imply that activated transcription factor Nrf2 and UPR are both implicated in the regulation of inflammation in BEECs.

Numerous studies concerning potential roles of UPR and possible UPR targets have been described recently [41,42]. ERS often leads to the accumulation of unfolded or misfolded proteins in the ER, thereby causing UPR. Therefore, characteristic molecules involved in UPR are commonly used as markers to indicate the occurrence of ERS. PERK, IRE-1, and ATF6 are important proteins in the signaling pathways during ERS [43] and numerous studies have indicated that the IRE1α-induced UPR pathway may contribute to inflammatory activation and brain injury [44,45]. Research has shown that inhibition of ERS-associated TXNIP/NLRP3 inflammasome activation by melatonin could alleviate the injury of endometrial epithelial cells induced by LPS in mice [46]. Similarly, ERS was involved in the upregulation of IL-6 production induced by LPS in bovine endometrial stromal cells [47]. These observations are consistent with our results that showed that under conditions of ERS and inflammation, PERK is activated by dissociating from its molecular chaperone GRP78 and downstream eIF2α phosphorylation. This process leads to the termination of most protein synthesis in BEECs but activates the expression of ATF4, which in turn upregulates CHOP expression [48]. In our study, knockdown of Nrf2 alleviated LPS-induced ER stress, whereas activation of the protein promoted ERS and inflammation. Thus, we hypothesize that Nrf2 may promote the inflammatory response through ERS in BEECs.

Intracellular Ca^2+^ mobilization is a key signaling event during the course of the inflammatory response [49]. The ER and mitochondria are the main sites of Ca^2+^ storage [50]. Ca^2+^ ions are critical during the inflammatory response and the ER is a crucial source of Ca^2+^ ions for this process. During inflammation, immune cells, including macrophages and neutrophils, release cytokines and chemokines that signal other immune cells to migrate to the site of infection or injury [51]. This process requires an increase in intracellular Ca^2+^ ion concentrations, which is mediated by the ER. Upon activation of immune cells, the ER releases Ca^2+^ ions into the cytosol through specialized inositol triphosphate receptor channels and ryanodine receptors [52]. This increase in cytosolic Ca^2+^ ions activates several downstream signaling pathways that promote the inflammatory response, including the activation of nuclear factor-kappa B (NF-κB, p-65) and the production of ROS [53]. However, excessive ER Ca^2+^ release may also lead to ERS and the activation of UPR, which may further exacerbate inflammation and contribute to tissue damage [53]. Research has shown that mitochondrial dysfunction and ERS in endometritis result from cytosolic Ca^2+^ overload [10]. Consistently, our results showed excessive Ca^2+^ release from the ER to the cytoplasm in LPS-treated cells compared with control BEECs. This imbalance in Ca^2+^ concentrations is one of the main reasons for the induction of ERS.

## 4. Materials and Methods

### 4.1. Bovine Uterine Collection and Cell Culture

All uterine samples in the present study were harvested from Holstein–Friesian dairy cows. Infertile cows were selected and animals with mastitis, hoof disease, or other diseases were excluded. Fresh uteri were collected from slaughterhouses and transported to the laboratory on ice within two hours. The uterine tissue was divided into a section that was frozen in liquid nitrogen for extraction of RNA and protein and a segment that was fixed in paraformaldehyde (4%) for HE staining and immunohistochemical testing. BEECs were isolated from healthy cornua uteri and were cultured in DMEM supplemented with penicillin (100 mg/mL) and streptomycin (100 U/mL) with 10% FBS at 37 °C in a humidified atmosphere with 5% CO_2_. The animal study protocol was approved by the Institutional Review Board of Northwest A&F University (protocol code: 2018ZX04002032; date of approval: 6 April 2021).

### 4.2. Total RNA Extraction and Real-Time Quantitative PCR

Total RNA in both uterine tissue and BEECs was extracted with the AG RNAex Pro Reagent (AG Bio, Changsha, China) and was reverse-transcribed into cDNA using the Evo M-MLV RT Kit (AG Bio) following the manufacturer’s protocol. Quantitative real-time PCR (qRT-PCR) was conducted using an SYBR qPCR Master Mix (Vazyme, Nanjing, China) at 95 °C for 2 min, followed by 40 cycles at 95 °C for 10 s and 60 °C for 30 s. The primer sequences used are shown in Table 1. The fold change of mRNA expression to *β-actin* was calculated using the 2^−△△Ct^ method.

### 4.3. Western Blot Analysis

Western blot analysis was conducted according to previously described procedures [54] with antibodies against the following proteins: GRP78 (Abcam, Cambridge, UK), P-IRE1 (Abcam), p-eIF2α (Abways Techology, Shanghai, China), PERK (Santa, Shanghai, China), Nrf2 (Proteintech, Wuhan, China), ATF4 (Santa), ATF6 (Abcam), *β-actin* (Proteintech), HRP-labeled goat anti-rabbit (ZHHC, Xi’an, China) or goat anti-mouse (ZHHC) immunoglobulin, Caspase3 (Abways Techology), CHOP (Abmart, Shanghai, China), or p65 (Abcam). Grayscale analysis was performed using Image J software (Version 1.8.0; Bethesda, Rockville, MD, USA).

### 4.4. Hematoxylin–Eosin Stain

Dairy cow uteri fixed in 4% paraformaldehyde were stained with the HE Stain Kit (Solarbio, Beijing, China) according to procedures described previously [10]. The morphology and inflammatory infiltration of the endometrial epithelium were evaluated using a Ni-U microscope (Nikon, Tokyo, Japan) by a single experienced observer who was blinded to the origins of the samples. Uteri were designated as healthy or endometriotic according to the levels of inflammatory cell infiltration and the expression of inflammatory cytokines.

### 4.5. Immunohistochemistry

The uterine expression of Nrf2 was assessed by immunohistochemical staining as described previously [10]. Briefly, samples were stained with DAB chromogenic solution (Maixin-Biotech, Fuzhou, China) and Nrf2 expression in healthy and endometriotic samples was evaluated with a microscope (Olympus, Tokyo, Japan).

### 4.6. Immunofluorescence Staining

BEECs were seeded in 6-well plates at 2 × 10^5^ cells per well and were subjected to the treatments indicated in the Results section. BEECs were subsequently fixed with paraformaldehyde (4%) for 30 min at room temperature, permeabilized with Triton X-100 (0.3%) for 15 min, and blocked with 5% FBS for 2 h. The samples were incubated overnight with p65 primary antibody at 4 °C followed by secondary antibody for 2 h at room temperature. Nuclei were stained with DAPI (Byotim, Shanghai, China) for 15 min at room temperature and were imaged with an A1Rsi confocal microscope (Nikon).

### 4.7. RNA Interference

BEECs were cultured in 6-well plates and transfected when the cell confluence reached 70–80%. Cells were washed twice with DMEM medium without fetal calf serum. The transfection of siRNA into BEECs was performed with TurboFect™ Transfection Reagent (ThermoFisher, Waltham, MA, USA) for 24 h. Subsequently, the cells were harvested and further experiments were performed. The sequences of the siNC control and siNrf2 target sequences were designed and synthesized by Genepharma, Shanghai, China (Table 2).

### 4.8. Flow Cytometry

Cell cycle progression was monitored with a Cell Cycle Detection Kit (Beyotime, Shanghai, China). Transfected cells were harvested and stained with annexin V/PI according to the manufacturer’s instructions. Briefly, 1 mL of ice-bath-cooled 70% ethanol was added to transfected cells, and the sample was mixed and fixed at 4 °C overnight. Cells were harvested by centrifugation at 1000× *g* for 5 min, the supernatant was discarded, and 1 mL of 70% ethanol was added to resuspend the cells. The sample was centrifuged, the supernatant was discarded, and cells were stained with annexin V/PI (Dye buffer 500 μL, Propyl iodide staining solution 25 μL, RNase A 10 μL). The results were analyzed with FlowJo software (1.53a/Java 1.8.0_112, NIH, Bethesda, MD, USA).

### 4.9. Cell Toxicity Assay

The cytotoxicity of TBHQ on BEECs was measured using Cell Counting Kit 8 (CCK-8, Beyotime, Shanghai, China). Briefly, BEECs were seeded in 96 wells (5 × 10^3^ cells/well) and were treated with various concentrations. After cultivating for different times, cells were treated with 10 μL of CCK-8 solution and incubated for 1 h at 37 °C. The absorbance of the samples was determined at 450 nm.

### 4.10. Intracellular Ca^2+^ Detection

The Fluo-4 Calcium Assay Kit (Beyotime) was used to detect intracellular Ca^2+^. Briefly, BEECs were seeded into 24-well plates and grown to 70% confluency. The cells were transfected with siNC or siNrf2 and treated with TBHQ (20 μM) and LPS. The BEECs were washed twice with sterile PBS and Fluo-4 Staining Solution (Fluo-4 AM 0.5 μL, Solubility Enhancer 0.5, Assay Buffer 249) was added. The cells were incubated at 37 °C for 30 min and washed with pre-cooled PBS twice. Emission from the Ca^2+^-bound Fluo-4 dye was observed with an A1Rsi confocal microscope.

### 4.11. Apoptosis Analysis

Apoptosis was detected with the Apoptosis Detection Kit with Mito-Tracker Red CMXRos and Annexin V-FITC (Beyotome) according to the manufacturer’s instructions. Briefly, BEECs were plated in 24-well plates at a density of 2 × 10^5^/mL and were subjected to the treatments indicated in the Results section. The culture medium was removed and cells were washed twice with sterile PBS. Annexin V-FITC binding solution (188 μL) was added followed by Annexin V-FITC (5 μL) with gentle mixing. Mito-Tracker Red CMXRos dye solution (2 μL) and Hoechst 33342 dye solution (5 μL) were added and mixed gently. Cells were incubated at 20–25 °C for 20–30 min without light and then placed on ice. The Mito-Tracker Red CMXRos fluoresces red, Annexin V-FITC fluoresces green, and Hoechst 33342 fluoresces blue when observed by laser confocal microscopy using a confocal microscope (Nikon A1Rsi, Tokyo, Japan).

### 4.12. Statistical Analysis

Results are reported as the arithmetic means ± SEM of three samples. The unpaired Student’s *t*-test was used to compare the results of healthy and endometritis samples, as well as of cells subjected to different treatment protocols. One-way ANOVA followed by Tukey’s post hoc test and Fisher’s LSD were used for multiple comparisons. A *p*-value <0.05 was considered statistically significant (* *p* < 0.05, ** *p* < 0.01, *** *p* < 0.001, **** *p* < 0.0001).

## 5. Conclusions

The Nrf2 transcription factor is expressed mainly in the luminal epithelium and glandular epithelium in the bovine uterus, and Nrf2 and ERS are activated during endometritis. Nrf2 promoted the inflammatory response by activating the PERK pathway during ERS to induce apoptosis of BEECs. In view of these observations, Nrf2 may be a viable target for the treatment of dairy cow endometritis.

## Figures and Tables

**Table 1:** Primer sequences for RT-qPCR.

Gene	ID	Sequences (5′-3′)	Product Size (bp)
*β-actin*	NM_173979.3	F: CCGCAACCAGTTCGCCAT R: CCCACGTACGAGTCCTTCTG	180
*TNF-α*	NM_173966.3	F: CTCCTTCCTCCTGGTTGCAG R: CACCTGGGGACTGCTCTTC	92
*IL-6*	NM_173923.2	F: CAGATCCTGAAGCAAAAGATCGC R: CCCACTCGTTTGAAGACTGC	91
*IL-8*	NM_173925.2	F: CATTCCACACCTTTCCACCC R: AGGCAGACCTCGTTTCCATT	116
*Nrf2*	XM_005202312.4	F: ATTCAAGTGCCACAGTAA R: AAAGTAGCAGAGGAGGG	100
*GRP78*	NM_001075148.1	F: GTGCCCACCAAGAAGTCTCA R: GTCAGGGGTCGTTCACCTTC	92
*PERK*	NM_001098086.1	F: CACAGGGACCTCAAGCCTTC R: TCCTCGTCTTGGTCCATTGC	98
*ATF4*	NM_001034342.2	F: GCTTAAGCCATGGCGCTTTT R: ATGTTGCGAGGTTTTGGTGC	111
*ATF6*	XM_024989877.1	F: TACTTCCAGCAGCACCCAAG R: GCACCACGGTCTGACCTTTA	150
*eIF2α*	NM_175813.2	F: ACCACCCTGGAGAGAACAGA R: GTGACCACTTTGGGCTCCAT	119
*IRE1*	XM_024980955.1	F: GCCATGAGGAATAAGAAGCACC R: TGGCATGGTAGGTGTGTGAG	136

**Table 2:** Oligos for siRNA.

Gene	Sequence (5′ to 3′)
si-NC	F: UUCUCCGAACGUGUCACGUTT R: ACGUGACACGUUCGGAGAATT
Si-*Nrf2*-1	F: GCCCAUUGAUCUCUCUGAUTT R: AUCAGAGAGAUCAAUGGGCTT
Si-*Nrf2*-2	F: GGAGCAAGAUUUAGAUCAUTT R: AUGAUCUAAAUCUUGCUCCTT
Si-*Nrf2*-3	F: GAGGCCAGAUAUUAAGAAATT R: UUUCUUAAUAUCUGGCCUCTT

## Data Availability

Due to data confidentiality requirements, the manuscript cannot provide raw data.
